# Novel genome sequences and evolutionary dynamics of the North American anopheline species *Anopheles freeborni*, *Anopheles crucians*, *Anopheles quadrimaculatus*, and *Anopheles albimanus*

**DOI:** 10.1093/g3journal/jkac284

**Published:** 2022-11-15

**Authors:** Cory Henderson, Karen Kemirembe, Sage McKeand, Christina Bergey, Jason L Rasgon

**Affiliations:** Department of Entomology, The Pennsylvania State University, University Park, PA 16801, USA; Department of Genetics, Rutgers University, New Brunswick, NJ 08901, USA; Department of Entomology, The Pennsylvania State University, University Park, PA 16801, USA; Department of Entomology, The Pennsylvania State University, University Park, PA 16801, USA; Department of Genetics, Rutgers University, New Brunswick, NJ 08901, USA; Department of Entomology, The Pennsylvania State University, University Park, PA 16801, USA

**Keywords:** *Anopheles*, evolutionary genomics, North American vectors, novel genome sequences

## Abstract

*Anopheles* mosquitoes are the principal vectors for malaria and lymphatic filariasis, and evidence for arboviral transmission under laboratory and natural contexts has been demonstrated. Vector management approaches require an understanding of the ecological, epidemiological, and biological contexts of the species in question, and increased interest in gene drive systems for vector control applications has resulted in an increased need for genome assemblies from understudied mosquito vector species. In this study, we present novel genome assemblies for *Anopheles crucians*, *Anopheles freeborni*, *Anopheles albimanus*, and *Anopheles quadrimaculatus* and examine the evolutionary relationship between these species. We identified 790 shared single-copy orthologs between the newly sequenced genomes and created a phylogeny using 673 of the orthologs, identifying 289 orthologs with evidence for positive selection on at least 1 branch of the phylogeny. Gene ontology terms such as calcium ion signaling, histone binding, and protein acetylation identified as being biased in the set of selected genes. These novel genome sequences will be useful in developing our understanding of the diverse biological traits that drive vectorial capacity in anophelines.

## Introduction

Vectors are living organisms that can transmit disease-causing agents between vertebrate hosts. Mosquitoes transmit disease-causing pathogens during the act of bloodfeeding, which is required by many species for reproduction (Franz *et al.* 2015). Vector-borne pathogens generally fall into the broad categories of parasites, bacteria, or viruses and result in over 700,000 deaths per year, primarily in tropical and subtropical regions and disproportionately affecting the poorest populations residing in these areas (WHO 2021).

*Anopheles* mosquitoes are the principal vectors for malaria and can transmit pathogens such as lymphatic filariasis and O'nyong'nyong virus. Malaria historically impacted the Americas but now the region has the lowest malaria endemicity in the world, though it still circulates within central and south America (Sinka *et al.* 2010). *Anopheles* mosquitoes do demonstrate limited evidence for viral transmission; in natural contexts, *Anopheles gambiae* and *Anopheles funestus* act as the primary transmitting vectors for O'nyong'nyong virus, and in laboratory contexts, multiple *Anopheles* species have demonstrated capacity for the transmission of Mayaro and Chikungunya virus (Yadav *et al.* 2003; Rezza *et al.* 2017; Brustolin *et al.* 2018).

Vector management approaches require an understanding of the ecological, epidemiological, and biological contexts of the vector species, and with the increase in interest and application of genetic strategies such as gene drives utilizing CRISPR-Cas9, the availability of genomic sequences of vector species is becoming an immensely important aspect of vector control. High-quality chromosome-level genome assemblies exist for certain model anopheline species such as *An. gambiae* and *Anopheles stephensi* (Holt *et al.* 2002; Miles *et al.* 2017; Chakraborty *et al.* 2021); however, most *Anopheles* species complexes are understudied and as such comprehensive comparative genomics studies on these species are difficult to perform. Currently, while native anopheline species ranges span the entirety of the continental United States, genome assemblies for many of these species are absent from the literature. The primary anopheline species in the United States are *Anopheles freeborni* in the west and *Anopheles quadrimaculatus* in the east, and members of the *Anopheles punctipennis* species group such as *Anopheles crucians* are also present throughout the eastern half of the country (Sinka *et al.* 2010). *Anopheles albimanus* is one of the primary anopheline mosquitoes in Mexico, Panama, and the Caribbean (Sinka *et al.* 2010) but does occur in parts of Florida as well (Pritchard *et al.* 1946).

In this study, we present novel genome assemblies for *An. crucians*, *An. freeborni*, *An. quadrimaculatus*, and *An. albimanus*. We examine the evolutionary relationship between these species and look for examples of selection occurring in shared single-copy orthologs identified within these novel genome assemblies. Of the 790 total single-copy orthologs shared between all the newly sequenced genomes, 289 demonstrate evidence for positive selection on at least 1 branch of the phylogeny constructed using the novel genome assemblies, with gene ontology terms such as calcium ion signaling, histone binding, and protein acetylation identified as being biased in the set of selected genes.

## Methods

### Species identification

Wild *Anopheles* larvae belonging to the species *An. crucians* and *An. freeborni* were provided to the Rasgon Vector Genetics Laboratory by collaborators at the Brunswick County Vector Control Agency (North Carolina, *An. crucians*) and the Benton County Mosquito Control Agency (Washington, *An. freeborni*). Larvae were shipped from their collection localities in whirl-paks (Whirl-Pak, Madison, WI, USA) in water from their collection source, at which point they were placed into pans and reared in the Millennium Sciences Complex Insectary (The Pennsylvania State University, University Park, PA, USA) at 27°C ± 1°C, 12 h light 12 h dark diurnal cycle at 80% relative humidity. Ground fish flakes (TetraMin, Melle, Germany) were used to feed larvae, and upon emergence adult mosquitoes were maintained with a 10% sucrose solution. *An. quadrimaculatus* (Orlando strain MRA-139) and *An. albimanus* (Stelca strain # MRA-126) were taken from colony populations maintained in the Millennium Sciences Complex Insectary initially provided by BEI Resources (Manassas, VA, USA).

Fourth instar larvae were isolated and identified using morphological features under light microscopy according to reference material for *Anopheles* larval identification in “Mosquitoes of Pennsylvania,” and species ranges of identifications were confirmed using ranges published in “Mosquitoes of New York” (Means 1987; Darsie and Hutchinson 2009). Larvae were reared separately after identification according to date of emergence and species. Following emergence species identification was then confirmed in adults by placing them at 4°C to force them into a state of paralysis, which was then maintained for species confirmation using morphological characteristics for adults as described in “Mosquitos of Pennsylvania” by placing individuals on a chill block under a light microscope (Darsie and Hutchinson 2009). Samples NC crucians 5–9 were originally identified as *An. punctipennis* by morphology, but mitochondrial assemblies place them more confidently as *An. crucians*.

### Genome assembly

DNA was extracted from individual mosquitos using the Qiagen DNEasy Blood and Tissue Kit with standard protocols. Extracted DNA underwent library preparation and genome sequencing at the New York University Langone Genome Technology Center (New York, NY, USA) using a NovaSeq 6000 with 150-bp paired-end sequencing to ∼30× coverage per sample. Raw sequencing reads had adapters trimmed and low-quality bases removed using Trimmomatic (v0.39) read trimming software with base settings (Bolger *et al.* 2014). Velvet (v 1.2.10) was run on trimmed and paired reads for each sample with k-mer length of 85 bp, minimum coverage of 5×, and expected coverage of 30× to generate the novel genome assemblies (Zerbino 2010). Velvet performs scaffolding and inserts Ns into genome assemblies to represent gaps between contigs. Velvet uses paired-end insert length for scaffolding which it estimates automatically. Genome completeness was measured using BUSCO (v 5.4.3) with the insecta_odb10 database to scan for complete conserved orthologs present within the novel assemblies (Simão *et al.* 2015). Finally, RepeatMasker (v 4.1.2.p1) was used to mask repetitive elements from the genome assemblies with the Anophelinae species setting and using Xs to signify masked repetitive elements (Smit *et al.* 1996). The repeat masked assemblies are not discussed in the text, but they are uploaded to GenBank with the nonmasked assemblies and gene predictions.

Mitochondrial genomes were assembled from interleaved raw sequencing reads for each sample using the mitoBIM (v1.9.1) pipeline (Hahn *et al.* 2013) and were then uploaded to BOLD systems to provide species identification for samples where ID was uncertain (Supplementary Table 1) (Ratnasingham and Hebert 2013). Augustus de novo gene prediction software was used to identify potential proteins within the novel assemblies using a training set of 5,876 full-length coding sequences from the *An. gambiae* PEST strain AgamP3 (GCA_000005575) genome assembly and functional prediction on the potential gene sets was achieved with InterProScan (v 5.55_88.0) (Stanke *et al.* 2008; Jones *et al.* 2014). Unless otherwise stated base settings were used.

### Phylogenetics and scanning for signatures of selection

To identify single-copy orthologs for use in creating a phylogeny for the novel genomes and for identifying signatures of selection, Exonerate (v2.4.0) protein2genome was run using proteins present in the *An. gambiae* PEST strain UP000007062 proteome published on uniProt against novel genome assemblies (Slater and Birney 2005; Bateman *et al.* 2021). Exonerate hits that had scores above 1,000, were represented at least 80% of full length in the novel genomes, were not duplicated in any genome, and were present in all genomes considered were kept for further analysis (Supplementary File 1 and Supplementary Table 2). Multiple sequence alignment (MSA) for all orthologs was generated using MUSCLE (v5.1) and pal2nal (14.1) was used to convert the MSA for each ortholog into an in-frame codon alignment (Edgar 2004; Suyama *et al.* 2006). Concatenated in-frame codon alignments for all orthologs were used to create a consensus phylogenetic tree using iqTree (2.2.0.3), and then individual gene trees were made for each ortholog and compared with the consensus tree to determine gene concordance and site concordance factors for each node of the consensus tree (Minh *et al.* 2020). HyPhy (v 2.5.41) software was used to run adaptive Branch-Site Random Effects Likelihood (aBSREL) to identify if positive selection occurred along any specific proportion of branches for the orthologs in question (Smith *et al.* 2015). Genes identified as having been selected for by aBSREL along any branch or subset of branches were analyzed for gene ontology term overrepresentation using topGO (v2.48.0) in R using the set of identified single-copy orthologs as the background. Unless otherwise stated base settings were used.

## Results and discussion

### Genome assembly

We created novel genome assemblies from wild-collected *An. crucians* and *An. freeborni* and from colony populations of *An. quadrimaculatus* and *An*. *albimanus.* For the natural samples representing *An. crucians*, we collected whole genome sequences from 6 individuals (NC crucians 2, 3, 4, 5, 6, and 8), and 2 *An. freeborni* were sequenced (WA freeborni 1 and 2) (Table 1). Of the colony *An. quadrimaculatus* and *An*. *albimanus*, 2 individuals each were sent for whole genome sequencing (COL albi 1 and 2 and COL quad 3 and 4).

**Table 1.** Information related to natural mosquito collections.

State	Molecular species ID	Specimen mumber	ID	Date received	Days posteclosion	DNA concentration (ng/μl − 100 μl total volume)	Collection GPS	Proportion of mosquito for DNA extraction
NC	*An. crucians*	2	NC crucians 2	2019 April 3	6	2.66	34.1697, −78.1688	1/10 mosquito
NC	*An. crucians*	3	NC crucians 3	2019 April 3	6	4.15	34.1697, −78.1688	1/10 mosquito
NC	*An. crucians*	4	NC crucians 4	2019 April 3	9	3.87	34.1697, −78.1688	1/10 mosquito
NC	*An. crucians*	5	NC crucians 5	2019 April 3	3	3.63	34.1697, −78.1688	1/10 mosquito
NC	*An. crucians*	6	NC crucians 6	2019 April 3	4	2.21	34.1697, −78.1688	1/10 mosquito
NC	*An. crucians*	8	NC crucians 8	2019 April 3	6	4.32	34.1697, −78.1688	1/10 mosquito
WA	*An. freeborni*	1	WA freeborni 1	2019 May 23	5	8.33	46.339903, −119.379405	Half mosquito
WA	*An. freeborni*	2	WA freeborni 2	2019 May 23	5	6.1	46.339903, −119.379405	Half mosquito
COLONY	*An. quadrimaculatus*	3	COL quad 3			40.15		Whole mosquito
COLONY	*An. quadrimaculatus*	4	COL quad 4			42.78		Whole mosquito
COLONY	*An. albimanus*	1	COL albi 1			17.41		Whole mosquito
COLONY	*An. albimanus*	2	COL albi 2			14.46		Whole mosquito

Table 2 displays genome sizes, N50, BUSCO scores, and other relevant information for all samples sequenced. *An. crucians* had the largest genome size of those sequenced in this study with 300.07 Mb average size per assembly, *An. freeborni* had an average genome size of 267.64 Mb, *An. quadrimaculatus* had an average genome size of 227.01 Mb, and *An. albimanus* had the smallest genome size of those sampled with an average assembly size of 169.29 Mb. N50 was highest for colony sourced samples, which is likely an artifact of lower genetic diversity within colony populations vs natural populations, ranging from 213.9 kb for an *An. albimanus* sample to 26.1 kb for an *An. crucians* sample. *An. albimanus* is the only species represented in this study that already has a genome assembly published, and they report a genome size of 170.85 Mb (Neafsey *et al.* 2015), comparable with our results. BUSCO scores suggest that all assemblies are relatively complete with between 91% and 97% single-copy ortholog representation from the BUSCO insecta_odb10 dataset (Simão *et al.* 2015).

**Table 2.** Genome sizes, N50, scaffold number, scaffold length, gap percentage, and BUSCO scores for all samples sequenced.

Sample	Species	Assembly size (Mb)	Scaffold N50 (kb)	Number of scaffolds	Max scaffold length (kb)	Gap percentage	Complete BUSCO percentage	Complete single-copy BUSCO percentage
COL albi 1	*An. albimanus*	169.64	213.965	17,092	861.783	0.22	97.4	96.1
COL albi 2	*An. albimanus*	168.95	203.749	15,451	1,145	0.19	97.3	96.1
COL quad 3	*An. quadrimaculatus*	226.513	112.34	33,592	1,133	0.46	94.9	92
COL quad 4	*An. quadrimaculatus*	227.51	101.16	36,258	1,133	0.45	94.1	91.2
NC crucians 2	*An. crucians*	300.308	32.636	114,926	436.406	1.3	94.7	91.9
NC crucians 3	*An. crucians*	296.783	33.191	118,270	442.04	1.34	94.2	91.4
NC crucians 4	*An. crucians*	299.908	34.41	117,474	794.975	1.44	93.7	91.4
NC crucians 5	*An. crucians*	301.547	26.505	116,962	568.616	1.23	94.9	92.9
NC crucians 6	*An. crucians*	300.772	34.958	116,170	791.158	1.41	94.7	92
NC crucians 8	*An. crucians*	301.102	38.365	114,523	471.009	1.45	94.6	91.7
WA freeborni 1	*An. freeborni*	264.892	53.703	115,231	538.994	1.17	94.2	93.1
WA freeborni 2	*An. freeborni*	270.397	57.001	115,503	518.202	1.24	94.9	93.8

A trend for high numbers of genes was observed in the natural genomes, with as many as 40,000 predicted for some *An. crucians* samples, and functional prediction using InterProScan determined many of these gene predictions to be classified as intrinsically disordered proteins (Jones *et al.* 2014). Furthermore, many of the genes identified as intrinsically disordered protein in the novel assemblies occurred as the beginning or end of a contig, and with the shorter contig lengths in natural vs colony sourced genomes. One possible explanation for the large number of poorly categorized proteins in the natural assemblies is that they are full-length proteins which have been fragmented by the lower quality of the assembly.

### Phylogenetics

**Fig. 1.**
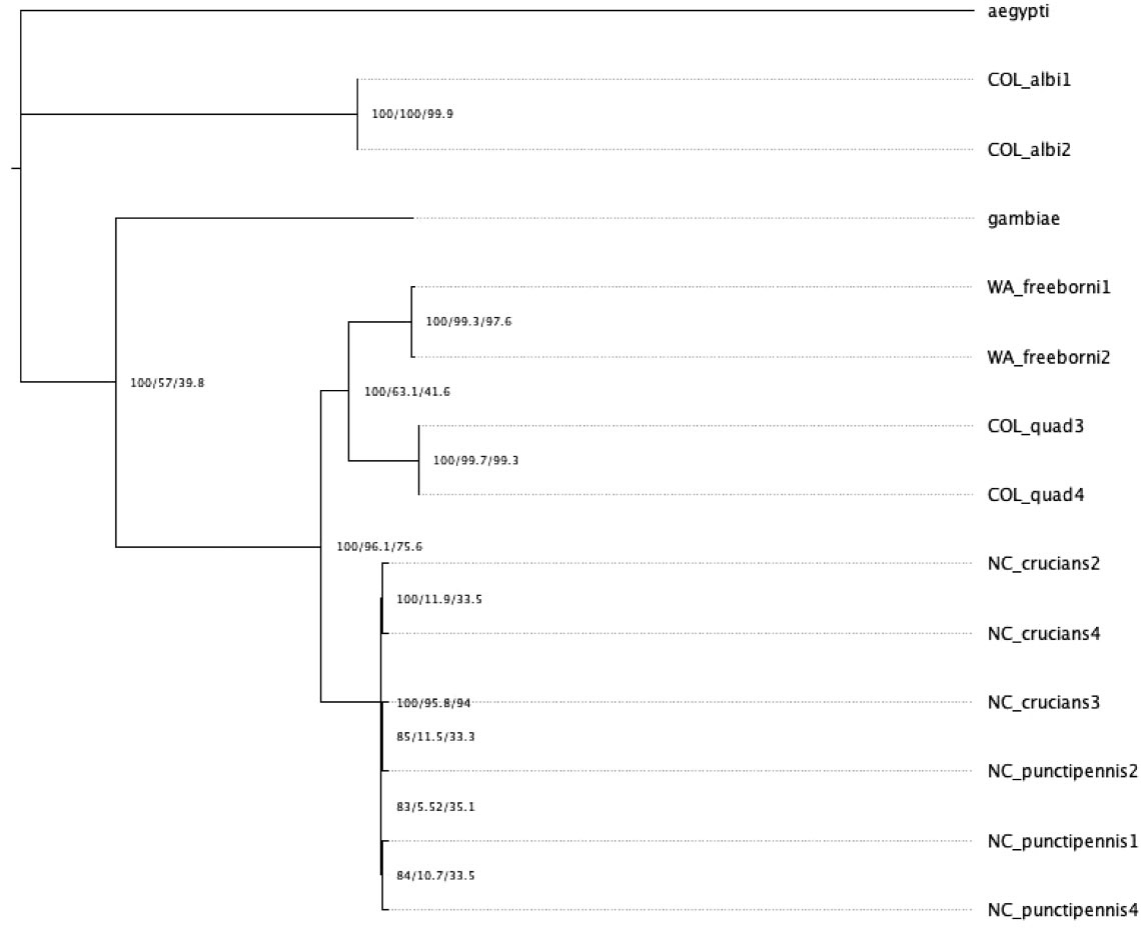
Maximum likelihood phylogenies with bootstrap values, gene concordance factor, and site concordance factors displayed on corresponding nodes in parentheses (i.e. each node contains bootstrap values/gene concordance factors/site concordance factors). We highlight here samples initially identified as punctipennis that grouped with crucians.

A total of 790 single-copy orthologs from the *An. gambiae* PEST strain proteome on UniProt were identified that were shared between all novel genome assemblies (Supplementary File 1) (Bateman *et al.* 2021). A maximum likelihood phylogeny was created using a concatenated alignment for a selection of 673 orthologs that were shared with *Aedes aegypti*, which acted as an outgroup. Individual trees were made for each ortholog for the calculation of site and gene concordance factors using the concatenated maximum likelihood phylogeny as a reference (Fig. 1).

*An. albimanus* and *An. gambiae* are placed on their own independent branches, while *An. freeborni*, *An. quadrimaculatus*, and *An. crucians/punctipennis* are on a branch together with 100% bootstrap support. *An. freeborni* and *An. quadrimaculatus* diverged with 100% bootstrap support into independent branches, while *An. crucians/punctipennis* diverges into its own branch with 100% bootstrap support; however, the topology within this branch was not well supported. There are nodes within the branch that makes the *An. crucians/punctipennis* portion of the phylogeny, each containing 2 samples with 2 gene concordance factors of 11.94, 11.49, and 10.75.

**Fig. 2.**
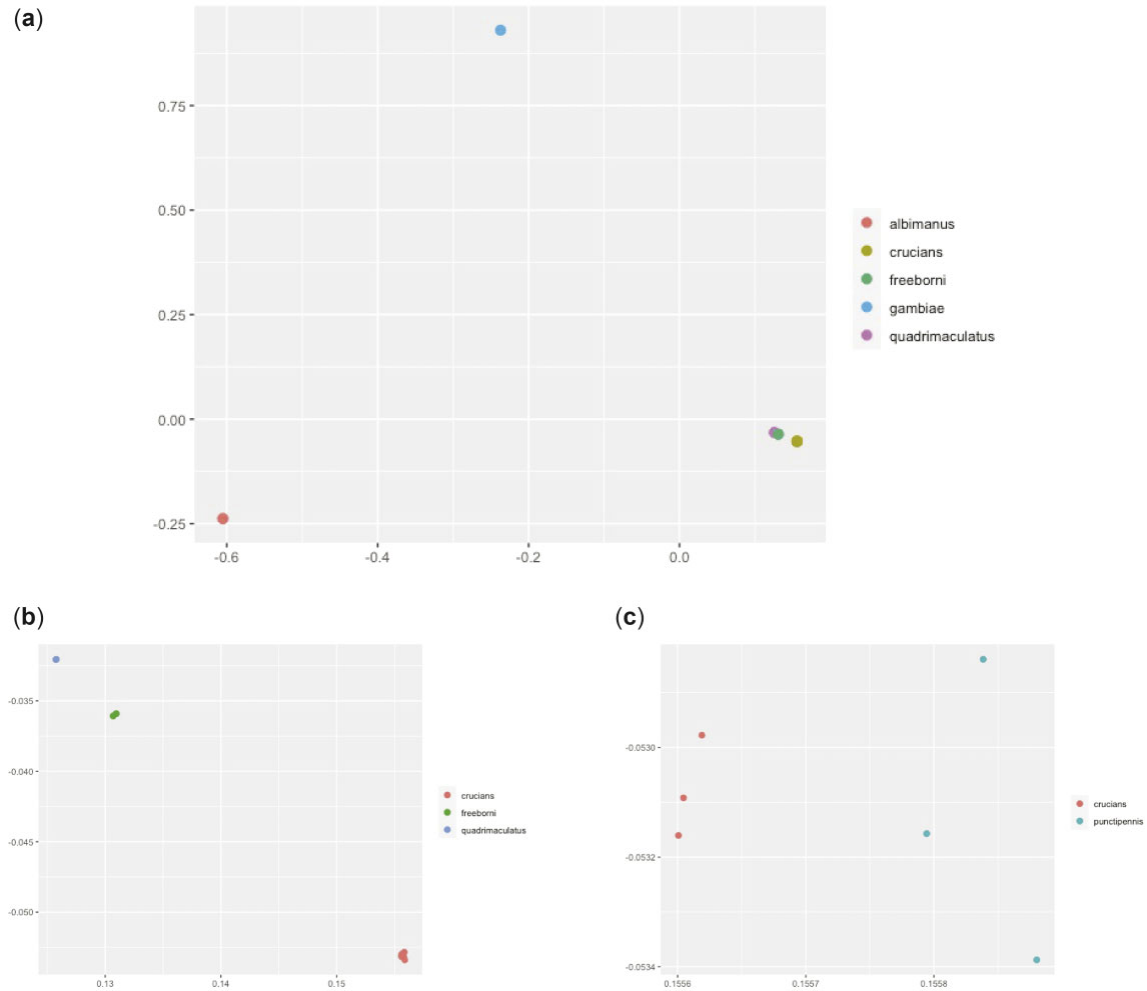
PCA of variants in the 790 identified single-copy orthologs shared between all novel assemblies and the *An. gambiae* PEST genome assembly and color coded by species. Image (a) shows all samples, image (b) isolates the more closely related group of *An. quadrimaculatus*, *An. crucians*, and *An. freeborni*, while image (c) isolates and delineates between the morphological IDs for *An. crucians* and *An. punctipennis*.

Mitochondrial genomes were assembled from the raw sequencing data collected for each sample and to these genomes were compared to a database of cytochrome c oxidase subunit 1 sequences using BOLD systems to determine species identification (Table 3) (Ratnasingham and Hebert 2013). The potential for admixture was considered between *An. crucians* and *An. punctipennis* as this has been observed previously for *An. crucians* and *A*nopheles *bradleyi* (Kreutzer and Kitzmiller 1971), but analysis of genome variants with ADMIXTURE on the 6 samples identified as either *An. cruciians* or *An. punctipennis* with *K* values ranging from 2 to 4 and did not identify any evidence for hybridization between samples confidently place them into 2 groups along species lines (Supplementary Fig. 1) (Alexander and Lange 2011). Principal components analysis (PCA) on genomic variants identified within the ortholog set demonstrates that the *An. crucians* branch separates into 2 groups, but they cluster much closer together compared with the other species represented within this study (Fig. 2). The evidence suggests that instead the branch for *An. crucians* represents a single species, not 2 species or a combination of 2 species and hybridized individuals.

### Evidence for selection

*dN*/*dS*-based analyses were performed using HyPhy to analyze the signatures of selection in alignments of the full set of 790 single-copy orthologs identified in all newly sequenced samples with a reduced tree not containing the *Ae. aegypti* outgroup. Signatures of selection of orthologs along a proportion of branches within the phylogeny were identified using aBSREL (Smith *et al.* 2015). aBSREL found 289 unique orthologs selected for in at least 1 of the 19 nodes identified in the phylogeny (Supplementary Table 3). Gene ontology analysis using topGO on terms regulated in any particular branch identified genes involved with structural molecules selected for nodes leading to *An. albimanus* samples (Supplementary Table 4) (Alexa and Rahnenfuhrer 2021). Nodes leading to all samples but *An. crucians* are biased for terms related to RNA processing and regulation of cellular processes. *An. freeborni* nodes were biased for terms related to the binding of lipids and RNA, regulation of cellular processes, and structural molecules.

### Conclusion

Limiting the spread of vector-borne disease can be improved by a deep understanding of the biology of the vector species of interest, and increasing our understanding of vector genetics is essential for progress in eliminating vector-borne pathogens. Phylogenetics of novel genome assemblies for *An. freeborni*, *An. crucians*, *An. albimanus*, and *An. quadrimaculatus* corresponds well to current understanding taxonomy. *An. freeborni, An. quadrimaculatus*, and *An. crucians* belong to *Anopheles* series of *Anopheles* subgenus; *An. gambiae—Cellia* subgenus; *An. albimanus—Nyssorhynchus* subgenus—basal divergences correspond well to subgenera. Closer relation of *An. freeborni* and *An. quadrimaculatus* also makes sense—these belong to *Maculipennis* Group (lower level taxonomy unit). Scanning for signatures of selection shows a broad set of terms being selected for along different branches of the phylogeny. These novel genome sequences will be useful in developing our understanding of the diverse biological traits that drive vectorial capacity in anophelines.

## Supplementary Material

jkac284_Supplementary_Data

## Data Availability

Genome sequences are available through NCBI under the reference numbers SAMN21013496, SAMN21013497, SAMN21013498, SAMN21013499, SAMN21013500, SAMN21013501, SAMN21013502, SAMN21013503, SAMN21013504, SAMN21013505, SAMN21013506, and SAMN21013507. Genome assemblies have been uploaded to figshare (https://doi.org/10.25387/g3.21136738: mitochondrial assemblies, genome annotations, functional predictions, and masked genome assemblies; https://doi.org/10.25387/g3.21277917: unmasked genome assemblies). Supplementary File 1 contains fasta sequences for the 790 orthologs used in the phylogenetic and hyPhy analyses. Supplementary Table 1 contains BLAST results against the mitochondrial genome assemblies. Supplementary Table 2 contains the UniProt and NCBI IDs corresponding to the orthologs used in the phylogenetic and hyPhy analyses. Supplementary Table 3 contains detailed output for hyPhy analysis. Supplementary Table 4 contains detailed results for topGO analysis on genes identified as being selected for in hyPhy analysis. Supplementary Fig. 1 demonstrates a phylogeny created using iqTree without *Ae. aegypti* as an outgroup, used for running hyPhy analysis. Supplemental material is available at G3 online.
