# Supplementary material for: Novel genome sequences and evolutionary dynamics of the North American anopheline species Anopheles freeborni, Anopheles crucians, Anopheles quadrimaculatus, and Anopheles albimanus
Source: G3 (Bethesda). 2022 Nov 15;13(1):jkac284. doi: 10.1093/g3journal/jkac284 (PMC9836346; doi:10.1093/g3journal/jkac284)
Supplement: jkac284_Supplementary_Data [file jkac284_supplementary_data.zip › Suppl/Supplemental_Figure_1_G3-2022-403782.pdf]

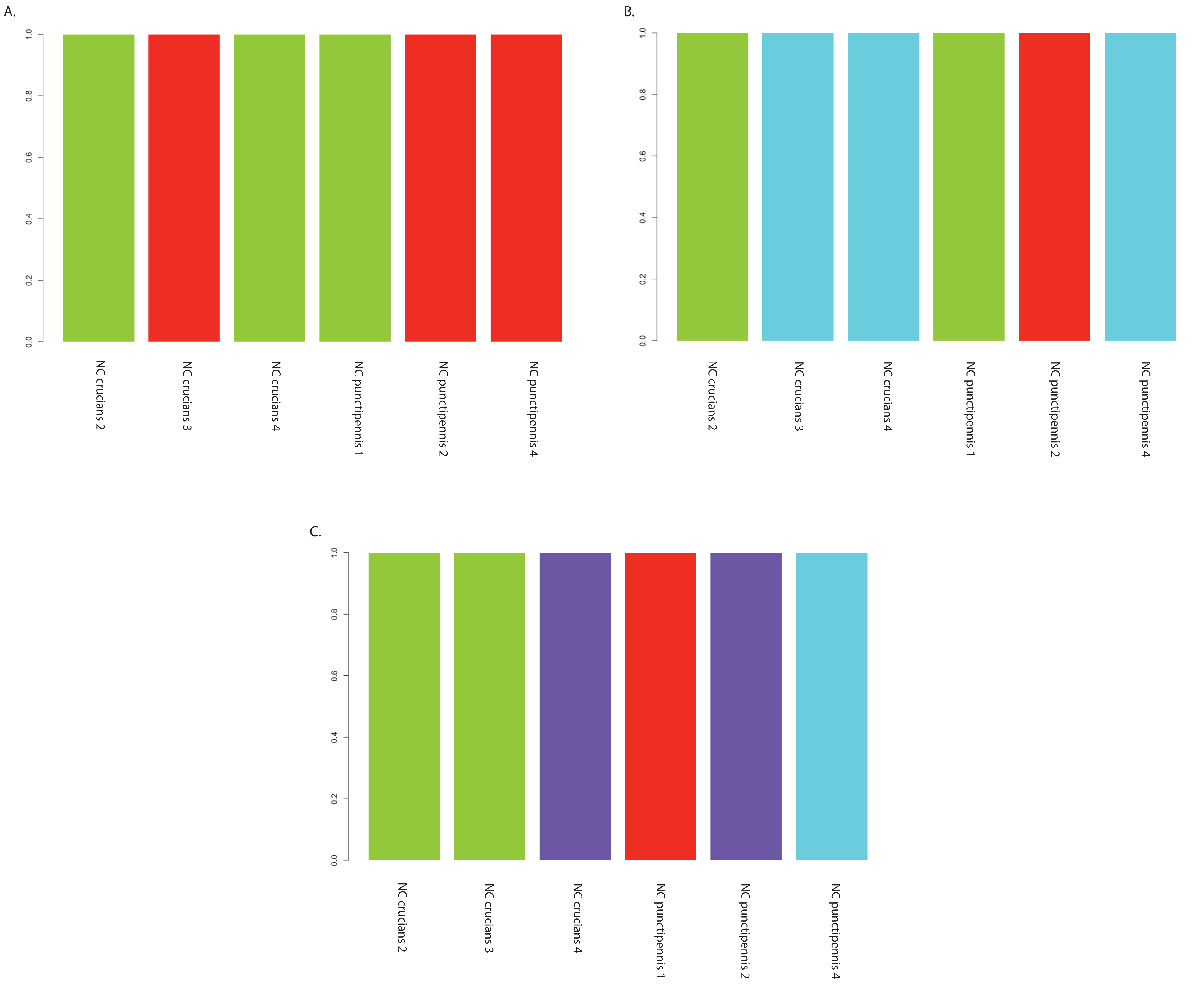

Supplementary Figure 1: Admixture plots created using ADMIXTURE software with inferred number of populations (K) equal to 2 (A), 3 (B), and 4 (C). Y-axis shows the proportion of a sample's genome from 0% to100% , X axis has the novel potential *Anopheles crucians/punctipennis* samples sequenced in this study, and the color of the bar for each samle represents the inferred populations representing that sample's genome. The color"s height on the Y axis represents the proportion of ancestry that population represents in the corresponding genome assembly.
