# Supplementary material for: Novel genome sequences and evolutionary dynamics of the North American anopheline species Anopheles freeborni, Anopheles crucians, Anopheles quadrimaculatus, and Anopheles albimanus
Source: G3 (Bethesda). 2022 Nov 15;13(1):jkac284. doi: 10.1093/g3journal/jkac284 (PMC9836346; doi:10.1093/g3journal/jkac284)
Supplement: jkac284_Supplementary_Data [file jkac284_supplementary_data.zip › Suppl/Supplemental_Material_G3-2022-403782.docx]

**Supplementary Materials Legends**

Supplementary Table 1: Results from BOLD systems query on mitochondrial assemblies.

Supplementary Table 2: Entrez and NCBI IDs for all identified shared single copy orthologs.

Supplementary Table 3: Results from HyPhy aBSREL analysis.

Supplementary Table 4: Results from gene ontology overrepresentation analysis on genes identified as selected by aBSREL using topGO.

Supplementary Figure 1: Admixture plots created using ADMIXTURE software with infer red number of populations (K) equal to 2 (A), 3 (B), and 4 (C). Y-axis shows the proportion of a sample’s genome from 0% to100%, X axis has the novel potential samples sequenced in this study, and the color of the bar for each sample represents the inferred populations representing that sample’s genome. The color ’’s height on the Y axis represents the proportion of ancestry that population represents in the corresponding genome assembly.

Supplementary File 1: Sequences used in multiple sequence alignment for all single copy orthologs identified. Ns represent gaps in alignment.
